# *CTC1* mutations in a Brazilian family with progeroid features and recurrent bone fractures

**DOI:** 10.1002/mgg3.495

**Published:** 2018-11-04

**Authors:** Forough Sargolzaeiaval, Jiaming Zhang, Jennifer Schleit, Davor Lessel, Christian Kubisch, Debora R. Precioso, David Sillence, Fuki M. Hisama, Michael Dorschner, George M. Martin, Junko Oshima

**Affiliations:** ^1^ Department of Pathology University of Washington Seattle Washington; ^2^ Institute of Human Genetics University Medical Center Hamburg‐Eppendorf Hamburg Germany; ^3^ Sarah Network of Rehabilitation Hospitals Belo Horizonte Brazil; ^4^ Discipline of Genetic Medicine Westmead Clinical School Sydney Faculty of Medicine and Health Westmead Australia; ^5^ Division of Medical Genetics Department of Medicine University of Washington Seattle Washington; ^6^ Department of Clinical Cell Biology and Medicine Graduate School of Medicine Chiba University Chiba Japan

**Keywords:** CTC1, genomic instability, Mendelian disorders, molecular genetics, progeroid syndrome, telomeres

## Abstract

**Background:**

Cerebroretinal microangiopathy with calcifications and cysts (CRMCC) is an autosomal recessive disorder caused by pathogenic variants of the conserved telomere maintenance component 1 (*CTC1*) gene. The CTC1 forms the telomeric capping complex, CST, which functions in telomere homeostasis and replication.

**Methods:**

A Brazilian pedigree and an Australian pedigree were referred to the International Registry of Werner Syndrome (Seattle, WA, USA), with clinical features of accelerated aging and recurrent bone fractures. Whole exome sequencing was performed to identify the genetic causes.

**Results:**

Whole exome sequencing of the Brazilian pedigree revealed compound heterozygous pathogenic variants in *CTC1*: a missense mutation (c.2959C>T, p.Arg987Trp) and a novel stop codon change (c.322C>T, p.Arg108*). The Australian patient carried two novel heterozygous *CTC1* variants, c.2916G>T, p.Val972Gly and c.2926G>T, p.Val976Phe within the same allele. Both heterozygous variants were inherited from the unaffected father, excluding the diagnosis of CRMCC in this pedigree. Cell biological studies demonstrated accumulation of double strand break foci in lymphoblastoid cell lines derived from the patients. Increased DSB foci were extended to non‐telomeric regions of the genome, in agreement with previous biochemical studies showing a preferential binding of CTC1 protein to GC‐rich sequences.

**Conclusion:**

*CTC1* pathogenic variants can present with unusual manifestations of progeria accompanied with recurrent bone fractures. Further studies are needed to elucidate the disease mechanism leading to the clinical presentation with intra‐familial variations of CRMCC.

## 1 INTRODUCTION

The *CTC1* gene (OMIM #613129), located on chromosome 17p13.1, encodes a 1,217 amino acid nuclear protein which is a component of the conserved telomere maintenance complex known as the CST complex (CTC1, STN1, and TEN1) (Rice & Skordalakes, 2016). The CST complex was initially proposed to play a role in telomere length homeostasis by reducing access of telomerase to telomeres in order to prevent excessive telomere lengthening (Chen, Redon, & Lingner, 2012). This was thought to result from the preferential binding of CST to the G strand 3′ overhang region of telomeres (Miyake et al., 2009). Subsequently, the CST complex was shown to promote telomere replication. The CTC1 protein was shown to form a heterodimer with the CST complex subunit, STN1, resulting in a DNA polymerase α accessory factor resembling Replication Protein A (RPA), which regulates telomere replication (Rice & Skordalakes, 2016). During the late S/G2 phase of the cell cycle, CST mediates the fill‐in synthesis of C‐strands (Feng, Hsu, Kasbek, Chaiken, & Price, 2017; Wang et al., 2012). There is increasing evidence suggesting genome‐wide roles of the CST complex during replication (Y. Wang et al., 2012). CST is capable of binding GC‐rich sequences throughout the genome (Chastain et al., 2016; Hom & Wuttke, 2017), where it may participate in resolving G‐quadruplex structures generated during replication stress (Bhattacharjee, Wang, Diao, & Price, 2017). This explains the global genome instability in hydroxyurea (HU)‐treated cells with CST pathogenic variants (Wang & Chai, 2018). Interestingly, telomere sequences and G4 structures are also substrates of the WRN helicase, null mutations at which are responsible for a segmental progeroid syndrome, the Werner syndrome (Croteau, Popuri, Opresko, & Bohr, 2014).

Diseases and conditions associated with pathogenic variants of *CTC1* include cerebroretinal microangiopathy with calcifications and cysts (CRMCC) and dyskeratosis congenita (DC). CRMCC, which is also called Coats plus syndrome, is a rare autosomal recessive disorder characterized by pre‐ and post‐natal growth restrictions, retinal telangiectasias, bilateral exudative retinopathies and intracranial calcifications, cerebral cysts, leukoencephalopathy, spasticity, ataxia, seizures, and dystonia (Anderson et al., 2012; Gu & Chang, 2013; Mansukhani et al., 2017; Polvi et al., 2012). It is known for its variable expressivity and can be associated with vascular ectasias of the gastrointestinal tract and premature features of aging such as severe osteoporosis, thin skin, graying of hair, and anemia. DC commonly manifests with a high rate of bone marrow failure, symptoms of premature aging, specific cutaneous features, and increased risk of developing cancer (Dokal, 2000; Keller et al., 2012; Walne et al., 2013).

In the first report of *CTC1* mutations in patients with CRMCC, all 14 patients examined were compound heterozygotes for a missense variant in one allele and a truncating variant in the other allele (Anderson et al., 2012). To date, the majority of *CTC1* pathogenic variants found in either CRMCC or DC are compound heterozygotes of a missense variant and a truncation variant, with a few exceptions of compound heterozygotes for two missense variants (Keller et al., 2012; Lin, Gong, Zhan, Wang, & Liu, 2017; Polvi et al., 2012; Walne et al., 2013). This suggested that combinations of two truncating mutations may result in intra‐uterine death because no patient has been found to carry two truncating mutations. Blood cells from CTC1 mutant patients were shown to exhibit shortened telomere lengths or telomere lengths at the lower range of normal (Anderson et al., 2012; Walne et al., 2013). One study, however, reported that there were no significant differences between the leukocyte telomere lengths of *CTC1* mutant patients and those of controls, raising the possibility that the disease mechanism of CTC1 mutations may involve non‐telomeric functions (Polvi et al., 2012).

We identified two pedigrees with progeroid features carrying novel *CTC1* variants among cases referred to the International Registry of Werner syndrome. Molecular and cell biological studies established the diagnosis of CRMCC in the Brazilian pedigree characterized by considerable phenotypic variability.

## 2 MATERIALS AND METHODS

### 2.1 Ethical compliance

The study was held in accordance with the Declaration of Helsinki protocol and approval of the Institutional Internal Review Board at the University of Washington.

### 2.2 Patient samples

The patients were referred to the International Registry of Werner Syndrome (http://www.wernersyndrome.org) for molecular diagnosis of their progeroid syndrome. Prior to the initiation of the study, written informed consent was given by the patient.

Upon arrival of the blood samples to the registry, genomic DNA was isolated from the patients’ blood samples and Epstein–Barr virus growth‐transformed lymphoblastoid cell lines (LCLs) were established from whole blood as previously described (Huang et al., 2006).

### 2.3 Exome sequencing and analysis

A library of DNA fragments was constructed and enriched for protein and RNA coding portions of the human genome using the Exome v1.0 (Integrated DNA Technologies) capture system. Paired‐end sequencing of the enriched library was performed using rapid run v2.0 (Illumina) chemistry on a HiSeq 2500 (Illumina) sequencer according to the manufacturer's recommended protocol. The resulting sequences were aligned to the human genome reference (hg19) using the Burrows–Wheeler Aligner (BWA) and variants identified with the Genome Analysis Tool Kit (GATK). Genbank accession number NG_032148.2 was used for analyses. Variants were annotated using an in‐house software tool based on SNPEff. Whole exome sequencing in Registry# MEAD1010 was performed as described previously (Lessel et al., 2017).

### 2.4 Western blot analyses and immunocytochemistry

Western blot analyses of CTC1 proteins in nuclear fractions of LCLs were performed using a commercial anti‐CTC1 antibody (Thermo Fisher Scientific, Cat.# PA5‐24695, Rockford, IL), with anti‐Lamin A/C (H‐110) antibody (Santa Cruz, Cat #sc20681, Dallas, TX) as an internal standard as previously described (Hisama et al., 2011).

Immunocytochemistry of 53BP1 was done following the protocol described previously, with some modifications (Saha, Cypro, Martin, & Oshima, 2014; Saha et al., 2013). LCLs were cultured on coverslips for two hours and then fixed with 4% paraformaldehyde in phosphate buffered saline (PBS; pH 7.4) plus 0.2% Triton X100 for 30 min at room temperature. Fixed cells on coverslips were incubated with primary antibody, rabbit anti‐53BP1 (NB100‐304SS, Novus Biologicals, Littleton, CO, USA) for 30 min at 37°C. They were then incubated with secondary antibody, Alexa Flour 488 donkey (Invitrogen Molecular Probes, Eugene, OR, USA) for 35 min at 37°C, and then post‐fixed with 4% paraformaldehyde for 20 min. After washing in PBS, coverslips were mounted on slides with DAPI (Vectashield, Vector Labs, Burlingame, CA) and then sealed with nail polish. Samples were co‐stained with the mouse anti‐TRF1 ([TRF‐78]ab10579, abcam, Cambridge, MA, USA) marker of telomeres as the primary antibody and Alexa flour 594 (Invitrogen Molecular Probes, Eugene, OR, USA) as the secondary antibody in co‐localization experiments.

### 2.5 Image processing and co‐localization studies

A Zeiss LSM 710 Confocal microscope and Zen software (Carl Zeiss) were used to obtain images of immunostained LCLs in *Z*‐stacks at the Keck center for microscopy (University of Washington, Seattle, WA, USA). Data for DNA damage foci in LCLs and co‐localization studies were analyzed using ImageJ software (https://imagej.nih.gov/ij/) (Saha et al., 2013, 2014). Correlations between 53BP1 expression and TRF1 were calculated using Pearson's co‐localization coefficients to express the intensity correlations of co‐localizing objects in each component of a dual‐color image (Dunn, Kamocka, & McDonald, 2011).

## 3 RESULTS

### 3.1 Clinical reports

**Figure 1:**
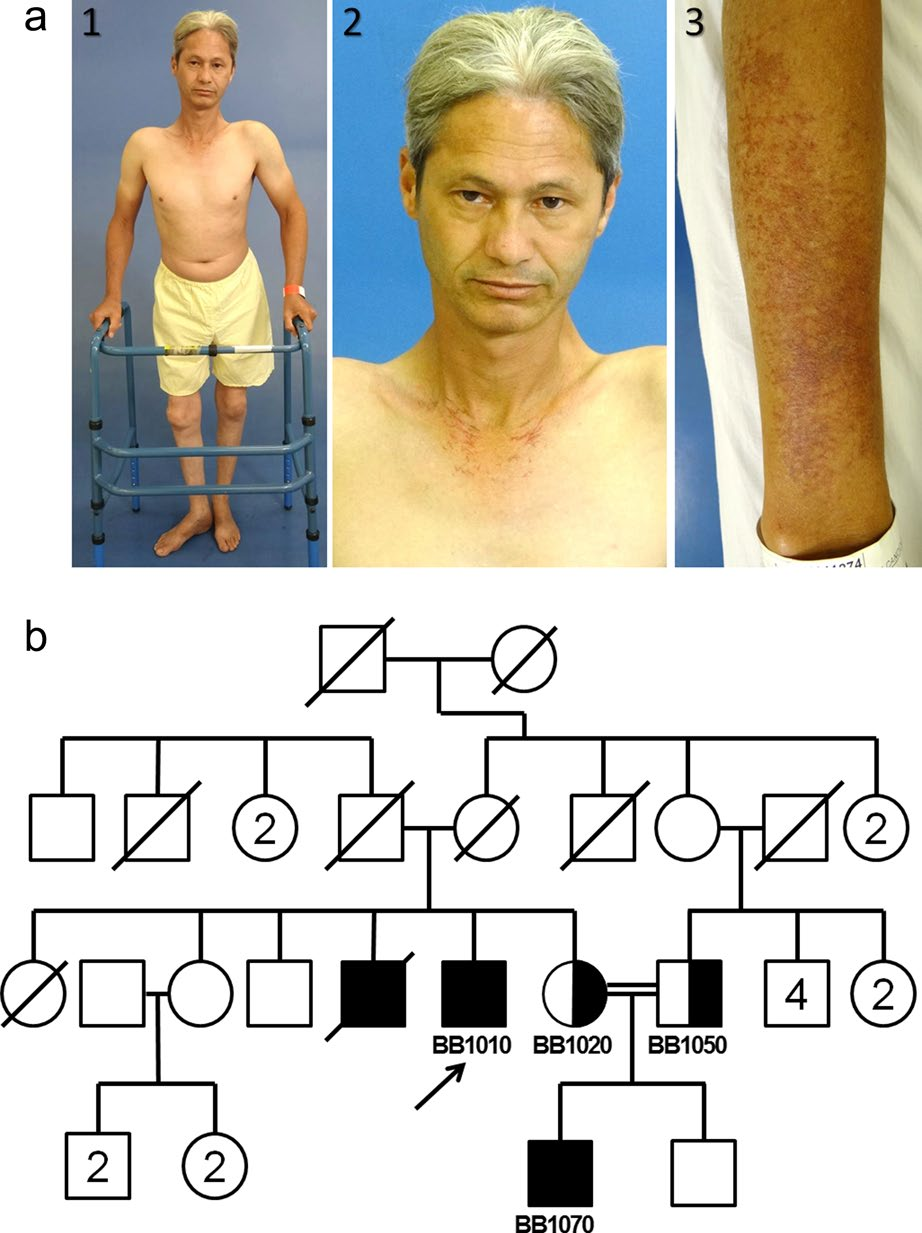
Profiles of Registry# BB1010. (a) Photos of the Registry# BB1010 showing short stature, height: 152 cm (1), premature graying of hair (2), and skin atrophy and hyperpigmentation in forearms (3). (b) Pedigree of BB1010

The index case, Registry# BB1010, is a 39‐year‐old Brazilian man (Figure 1) who was normal at birth and had normal development until 15 years of age, when he first began to exhibit progeroid features. During his teens, he gradually began to develop gray hair, atrophic skin, change in his voice, osteoporosis and short stature, features that are seen in other progeroid syndromes, including the classical Werner syndrome, a recessive segmental progeroid syndrome caused by null mutations of *WRN*, a member of the RECQ family of DNA helicases (Oshima, Martin, & Hisama, 2016). He also began to have multiple bone fractures, including those involving femurs, odontoids, costal arches, and thoracic and lumbar vertebrae. In addition, he was found to have hypogonadism at age 39, which was associated with normal testosterone and dehydroepiandrosterone levels and elevated luteinizing hormone. Neurologic examinations showed no focal neurological deficits, and ophthalmologic examinations showed no evidence of cataracts or retinopathy.

At the time of referral to the registry, his height was 152 cm (<1st %tile; *Z* score = −3.4), body weight was 39.9 kg (<1st %tile; *Z* score = −3.4), and he exhibited a generalized prematurely aged appearance. He was able to walk with the assistance of a walker. Telangiectasias of the neck and forearms were noted. Routine laboratory tests revealed a normochromic, normocytic anemia, normal fasting glucose and lipid panels, normal total and ionized calcium and normal levels of parathyroid hormone. An electrocardiogram revealed non‐specific changes in ventricular repolarization, although a chest X‐ray showed no evidence of cardiac hypertrophy or signs of pulmonary involvement. A brain MRI revealed an area of encephalomalacia in the left frontal lobe, which was attributed to previous head trauma. No intracranial calcifications were reported. The patient had a similarly affected brother (Registry# BB1030), who had had a brain MRI which revealed non‐specific cerebral white matter hyperintensities; he died of myocardial infarction at age 45. An autopsy was not performed.

The proband's nephew, Registry# BB1070, is an 18‐year‐old man with premature graying of hair beginning at age 9 years, short stature (height: 151 cm, *Z* score = −3.6), voice changes, and a unilateral cataract (left eye). Ophthalmologic examinations revealed retinal microangiopathy and retinal hemorrhages which were treated several times by laser photocoagulation. He had sparse facial hair and absent axillary hair. He had a single forearm fracture at age 8 years, after a fall, and exhibited osteopenia on skeletal X‐ray. He did not undergo brain imaging. Laboratory evaluations revealed a chronic anemia.

The mother and the father of BB1010 and BB1030 were unaffected. The mother of BB1070 (Registry# BB1020, sister of BB1010) and the father of BB1070 are first cousins (Figure 1b). The family reported no other consanguineous marriages.

The second case, Registry# MEAD1010, is a 49‐year‐old Australian woman who initially presented with stress fractures in her tibias and feet at 11 years of age. Later, she developed cutaneous striae and thinning of the skin in her late teenage years and a telangiectatic rash in sun‐exposed areas of skin in her 20s. At the time of referral to the registry, her height was 169.6 cm, (83.6%tile, *Z* score = −1.0), her weight was 60.5 kg (62%tile, *Z* score = −0.31), and she had evidence of mild premature aging of her facial features as compared to other family members. She had recurrent non‐healing stress fractures of long bones with relative sparing of the vertebral column, characteristic atrophic and tight skin, premature graying and thinning of hair, telangiectasias, and osteoporosis. She was diagnosed with progressive renal disease with evidence of diffuse mesangial sclerosis on biopsy and received a renal transplant at 47 years of age. Her parents (mother Registry# MEAD1060, 78‐year‐olds, and father Registry# MEAD1050, 78‐years‐old) were not known to have any health problems and did not have evidence of bone fragility.

### 3.2 Identification of *CTC1* pathogenic variants

**Figure 2:**
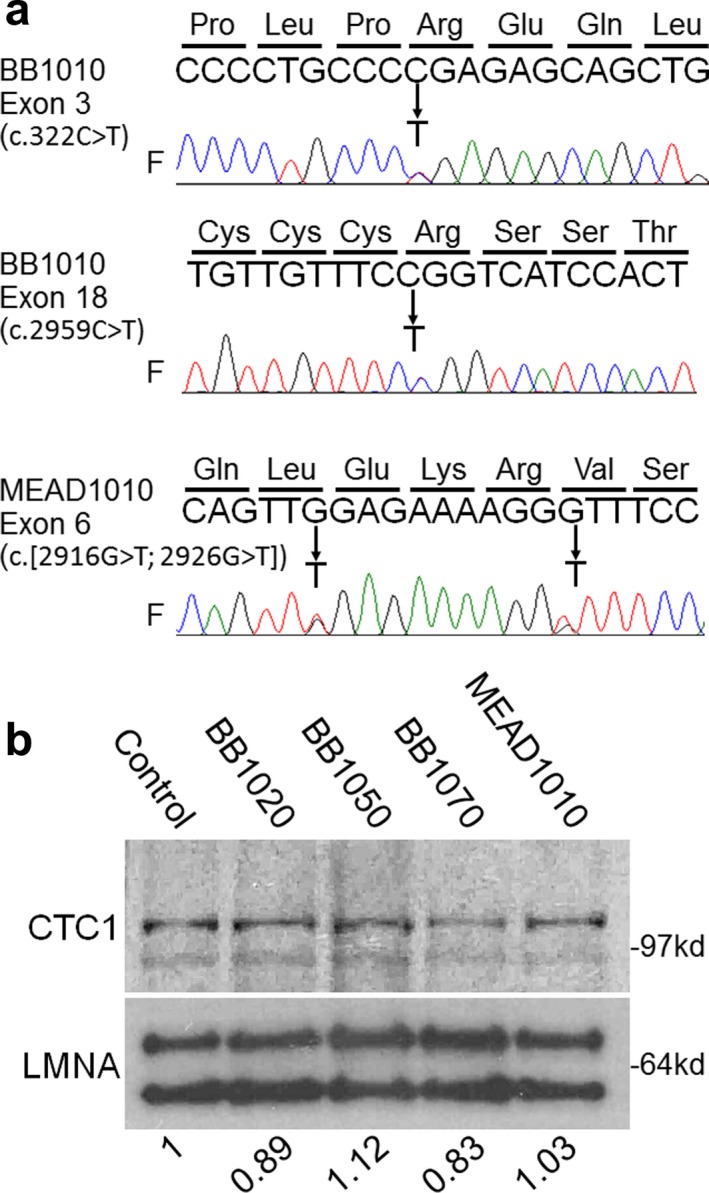
Identification of *CTC1* variants. (a) Sequencing results of *CTC1* exons. *CTC1* variants found in BB1010 and MEAD1010 are shown as follows: arrows indicate heterozygous changes. F indicates forward sequencing. (b) Western analysis of nuclear proteins in the BB1010 and MEAD pedigrees. The top panel shows the CTC1 protein, and the bottom panel shows the lamin A/C protein (LMNA). Numbers indicate the CTC1 protein levels relative to that of the control after normalization with the internal standard, lamin A/C

Whole exome sequencing was performed on DNAs isolated from blood samples from BB1010, BB1020, BB1050, and BB1070. Analysis revealed two heterozygous pathogenic variants in the *CTC1* of both affected individuals, BB1010 and BB1070. One variant, c.322C>T, introduces a premature stop codon, p. Arg108*, within exon 3, and the other variant, c.2959C>T, results in the substitution of arginine by tryptophan, p.Arg987Trp, in exon 18 (Figure 2a). The presence of these two heterozygous variants was also confirmed by Sanger sequencing in a sample from the proband's deceased brother, BB1030. BB1020 was heterozygous for c.2959C>T, p.Arg987Trp, and BB1050 was heterozygous for c.322C>T p.Arg108*. These results confirm the genetic diagnosis of Coats plus syndrome caused by biallelic variants of *CTC1*.

The variant c.2959C>T, p.Arg987Trp, has been reported in three compound heterozygous individuals with CRMCC (Anderson et al., 2012). This variant has also been reported in 16 heterozygous individuals in the gnomAD database (Genome Aggregation Consortium, gnomad.broadinstitute.org) with a population frequency of one in 8661 individuals, which is consistent with an autosomal recessive disorder. c.322C>T, p.Arg108* had not previously been reported in affected patients, but has been identified in one individual of Latino descent in the gnomAD database. The population frequency of this variant is approximately one in 16,791, which is also consistent with an autosomal recessive disorder. In addition, both variants have high phred‐scaled CADD (v1.3) scores (which take into account conservation and the scores from other pathogenicity predictors such as PolyPhen‐2 and SIFT), consistent with pathogenic recessive variants: 34.0 for p.Arg987Trp and 35.0 for p.Arg108*. Neither variant is reported in the homozygous state in gnomAD.

Two *CTC1* variants of unknown significance were found in another individual in our Registry, MEAD1010. MEAD1010 carried two novel heterozygous variants, c.2916G>T, p.Val972Gly and c.2926G>T, p.Val976Phe in exon 6 (Figure 2b). Phred‐scaled CADD scores were 24.1 for p.Val972Gly and 27.1 for p.Val976Phe. Sequencing analysis of parental samples showed that both variants were present in the father (c.[2916G>T; 2926G>T]) but neither were present in the mother, indicating that these heterozygous double changes in a single allele are unlikely to be responsible for the clinical features of MEAD1010.

Western blot analyses of nuclear proteins were performed in LCLs using an antibody against amino acid 767–795 of the *CTC1* protein. Either wild‐type or missense mutant CTC1 proteins with expected sizes of ~135kd were detected at similar levels in all samples (Figure 2b). We were unable to assess the expression levels of the truncation mutant *CTC1* protein due to the unavailability of an N‐terminal CTC1 antibody. These results suggest that there is a compensatory increase of CTC1 expression from the wild‐type or missense alleles in the cells with a truncated variant.

### 3.3 Accumulation of DNA damage in *CTC1* mutant cells

**Figure 3:**
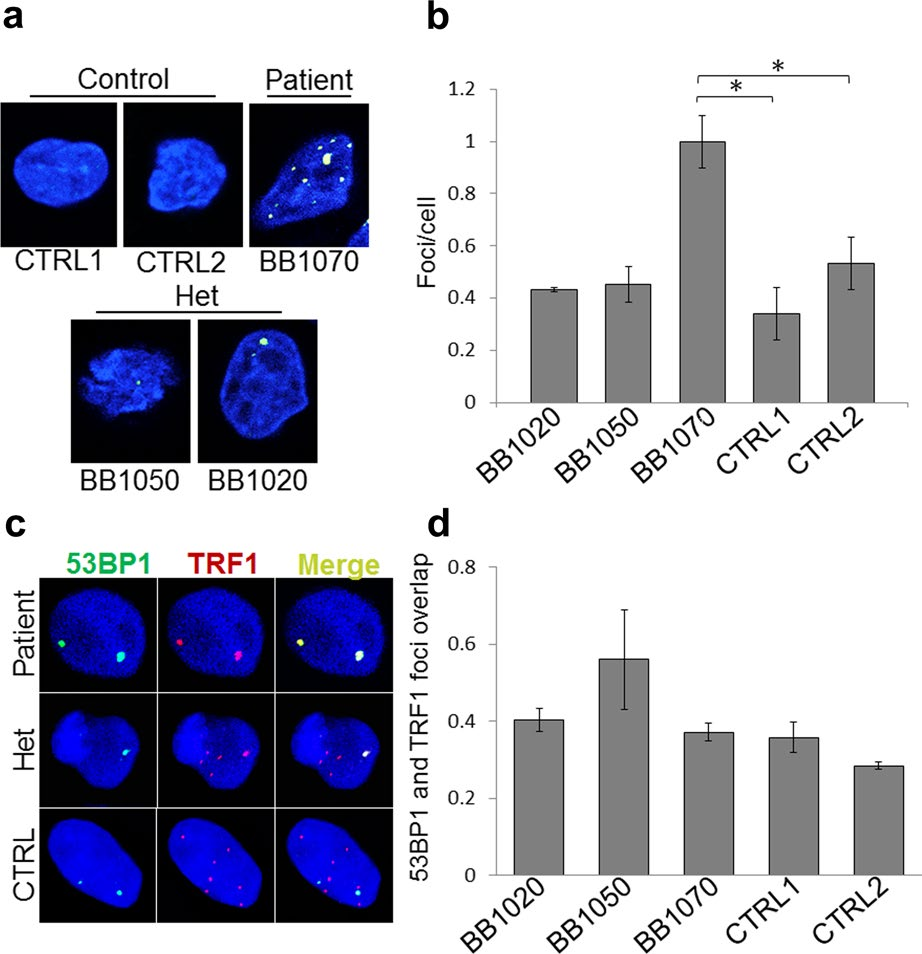
Accumulation of DNA damage foci in *CTC1* mutant LCLs. (a) Immunostaining of 53BP1 (green) counterstained with DAPI staining for DNA (blue). Representative pictures of cells from the patient, 2 heterozygotes, and 2 controls are shown. (b) Average number of 53BP1 foci per cell is calculated for each LCL. * indicates *p* < 0.05. A total of approximately 200 cells were analyzed for each experiment. (c) Sub‐nuclear localization of 53BP1 foci. Co‐immunostaining of 53BP1 (green), telomere binding protein, TRF1 (red), and DNA (using DAPI, blue) were done in control and *CTC1* mutant cells. (d) Correlation between 53BP1 and TRF1 signals. Calculated Pearson's Coefficients are shown as measures of the linear correlations between 53BP1 and TRF1 signals. Values are shown between +1 and −1; +1 for completely positive linear correlation and −1 for completely negative linear correlation. Approximately 100 cells were analyzed for each cell line. Het stands for heterozygous and CTRL stands for control

*CTC1* mutations alter the function of the CST complex, which may result in shortening of telomeres and DNA damage responses (Anderson et al., 2012; Huang, Jia, Chastain, Shiva, & Chai, 2017). Accumulation of DNA damage foci accompanied by increased p53 expression level and increased p53 activities was previously reported in *CTC1* deficient mouse embryos (Gu et al., 2012). Biological effects of *CTC1* variants in our Brazilian pedigree were assessed in LCLs by immunostaining of a DNA damage marker, 53BP1. The average number of 53BP1 foci per cell was increased by 2.8‐fold, from 0.38 in control 1 and 0.41 in control 2 to 1.09 in the patient, BB1070 (*p* = 0.048). In heterozygotes, there was a trend toward an increased number of foci, with 0.44 foci/cell in BB1020 (*p* = 0.126) and 0.50 in BB1050 (*p* = 0.107) which, however, were not statistically significant (Figure 3a).

In order to determine whether damage foci are localized to the telomeres or extended to non‐telomeric regions of the genome, we measured co‐localizations of 53BP1 and a telomere marker, TRF1. Co‐localization analysis, calculated by pixel intensity spatial correlation analysis (Dunn et al., 2011), revealed that the majority of DNA damage signals were not limited to telomeric DNA in all LCL groups. No significant differences were observed among the control, heterozygous, and compound heterozygous groups (Figure 3c). This finding indicates that genomic instability caused by aberrant CTC1 function is not limited to telomere regions but can also affect other regions of the genome.

## 4 DISCUSSION

In this study, we report two families with novel *CTC1* variants in patients referred to the International Registry of Werner Syndrome who were ascertained because of signs of accelerated aging. In a Brazilian pedigree (Registry# BB1010), all three affected individuals carried a novel heterozygous variant, c.322C>T, p. Arg108*, in exon 3 and a previously reported missense variant, c.2959C>T, p.Arg987Trp, in exon 18 (Anderson et al., 2012). This is consistent with a previous report that virtually all early‐onset CRMCC is compound heterozygotes of a truncation variant and a missense variant (Anderson et al., 2012; Gu & Chang, 2013). Although BB1020 and BB1050 are maternal first cousins and the parents of BB1070, they carried different variants. The family members denied any knowledge of a biological relationship between the fathers of BB1020 and BB1050. Given the rarity of the variants, however, it is quite possible that they are identical by descent at the *CTC1* locus.

We also identified an Australian case, MEAD1010, with novel heterozygous *CTC1* missense variants, c.2916G>T, p.Val972Gly and c.2926G>T, p.Val976Phe in exon 6. Analysis of the parental DNA revealed that these two variants were in cis (c.[2916G>T; 2926G>T]) and inherited from the apparently unaffected father, MEAD1050.

Despite the fact that the pathogenic variants found in the BB family were previously reported in CRMCC patients (Anderson et al., 2012), the clinical features of the three patients in the BB1010 pedigree were not typical. CRMCC patients with *CTC1* mutations typically develop symptoms during infancy or early childhood. Cerebral findings such as intracranial calcifications and cysts accompanied by retinal telangiectasias and other vascular abnormalities have been frequently observed in affected individuals. Our two studied patients, BB1010 and BB1070, had relatively late onsets of the disease and were lacking cerebral and retinal manifestations, which are diagnostic features of CRMCC. The different manifestations observed in the affected patients could be indicative of the clinical variability of this disease, a variability that could result from differences in genetic backgrounds and/or environmental factors. Moreover, recurrent bone fractures and poor bone healing were the most prominent features of the 39‐year‐old index case of our Brazilian pedigree. Skeletal abnormalities, including generalized osteopenia, altered longitudinal growth, and metaphyseal abnormalities, have been previously reported in CRMCC patients (Toiviainen‐Salo et al., 2011). It has been suggested that diseases that cause impaired telomerase function or shortening of telomeres may lead to an osteoporotic phenotype and age‐related bone loss (Saeed et al., 2011). It is unusual, however, to observe progeroid features together with recurrent bone fractures as the major presenting clinical features in *CTC1* compound heterozygous patients.

The LCLs established from the blood sample of patient BB1050 exhibited a 2.8‐fold increase of DNA damage foci, as assessed by immunostaining of 53BP1 (Figure 3a,b). This is similar to what has been reported in *CTC1* deficient mouse embryonic fibroblasts (Gu et al., 2012). Co‐immunostaining of 53BP1 and TRF1 showed that the correlations of the sub‐nuclear localizations of these proteins were not different among LCLs derived from the patient, heterozygous parents or controls, supporting the notion that genomic instability caused by aberrant CTC1 function is not limited to the telomeres, but extends to additional regions of the genome (Bhattacharjee et al., 2017; Y. Wang & Chai, 2018). Previous studies also indicate that that most of the *CTC1* missense mutations can form a CST complex with STN1‐TEN1 and display telomeric localizations (Gu & Chang, 2013).

Increased p53 expression and activity levels were seen in *CTC1* deficient mouse embryonic fibroblasts with increased DNA damage foci (Gu & Chang, 2013). We, however, did not observe an increase in p53 protein levels or p53 activity levels (as measured by p21 expression levels) in our *CTC1* mutant LCLs (Supporting Information Figure S1). This may be due to the Epstein–Barr virus immortalization used for the establishment of the LCLs, which alters telomere kinetics. In addition, it is important to consider different impacts of disease‐associated human *CTC1* mutations in mouse and human tissues. It has been shown in previous studies that one variation can differentially affect the function of CTC1 proteins in human mouse cells in terms of interactions with DNA polymerase α primase and binding STN1 to form the CST complex (Chen, Majersk, & Lingner, 2013). Unfortunately, further studies of these issues using primary fibroblasts were unable to be performed because of the unavailability of skin biopsies.

The International Registry of Werner Syndrome has identified a series of distinct genetic mutations responsible for a range of novel segmental progeroid syndromes among samples submitted to our International Registry from physicians all over the world in order to rule in or rule out a diagnosis of the Werner syndrome (Maezawa et al., 2018; Yokote et al., 2017). These cases were operationally classified as examples of “Atypical Werner Syndrome.” Subsequent studies of the responsible mutations revealed that, with a single exception (pedigrees with a novel Peruvian type 2 Berardinelli–Seip syndrome) (Purizaca‐Rosillo et al., 2017), all highlighted major roles for DNA damage repair and response pathway genes, including *LMNA* (nuclear structure and chromatin interaction) (Chen et al., 2003), *POLD1* (DNA polymerase delta) (Lessel et al., 2015), *SPRTN* (Lessel, Vaz, et al., 2014), *ERCC4* (nucleotide excision repair) (Mori et al., 2018), *MDM2* (major inhibitor of p53) (Lessel et al., 2017), *SAMHD1* (regulation of dNTP pools) (Lessel, Saha et al., 2014), and *CTC1*. The present study further supports the concept of genomic instability as a major mechanism of accelerated aging.
