# Supplementary material for: CTC1 mutations in a Brazilian family with progeroid features and recurrent bone fractures
Source: Mol Genet Genomic Med. 2018 Nov 4;6(6):1148–56. doi: 10.1002/mgg3.495 (PMC6305643; doi:10.1002/mgg3.495)
Supplement: Supplementary file 1 [file MGG3-6-1148-s001.pdf]

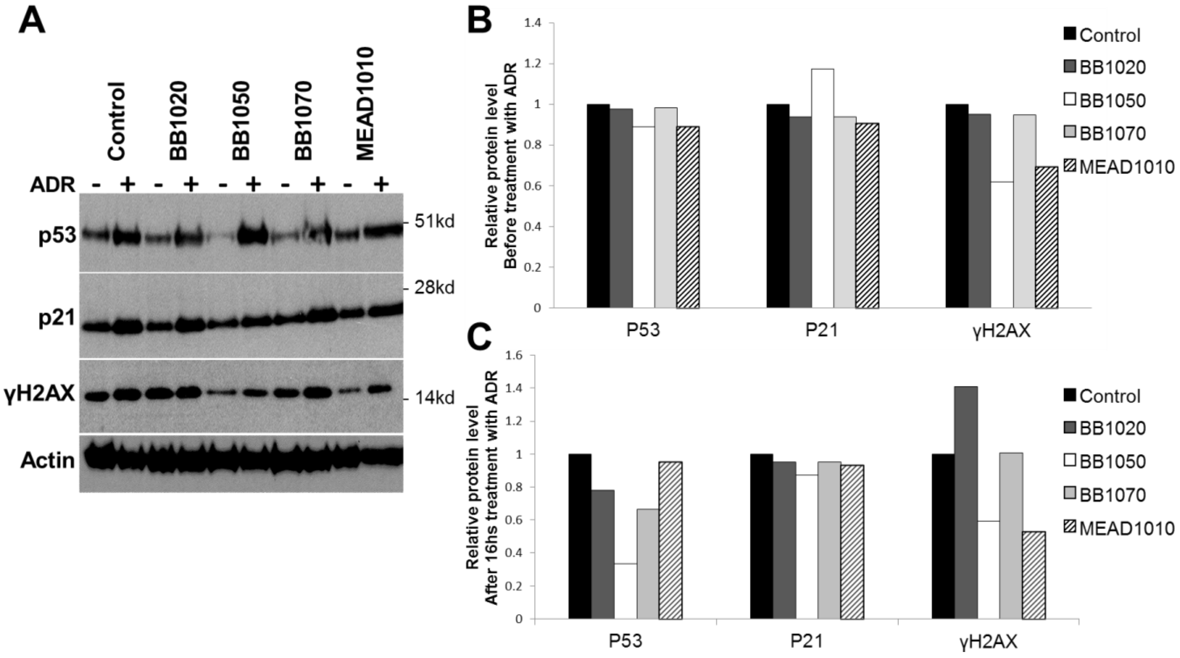

Supplementary Figure S1. Western blot analysis of p53, p21 and γH2AX. **(A)** LCLs derived from BB family members, MEAD1010 and control were treated with (+) or without (-) 3μM Adriamycin (ADR) for 16 hours. Overexposed autographies are shown for clarity. **(B)** Steady level of p53, p21 and γH2AX normalized with actin. **(C)** Responses to ADR were assessed by the ratio of protein levels before and after ADR treatment. No significant differences were observed between the expression levels of p53 and p21 proteins in *CTC1* mutant and normal cells before and after treatment with Adriamycin. Western analysis of p53, p21 and γH2AX were done using the commercial antibodies, Anti-p53 (Ab-6) mouse mAB (DO-1) (Calbiochem, Cat.# OP43L, Darmstadt, Germany), mouse monoclonal Anti-p21WAF1/Cip1 antibody (Sigma-Aldrich, Cat.# P1484, St. Louis, MO) and phosphor-histone H2A.X (Ser139) antibody (Cell Signaling, Cat.#5277, Danvers, MA)
